# Recognition of Foot Strike Pattern in Asian Recreational Runners

**DOI:** 10.3390/sports7060147

**Published:** 2019-06-17

**Authors:** Aurélien Patoz, Thibault Lussiana, Cyrille Gindre, Kim Hébert-Losier

**Affiliations:** 1 Research and Development Department, Volodalen Swiss SportLab, 1860 Aigle, Switzerland; Cyrille@volodalen.com; 2 Research and Development Department, Volodalen, 39134 Chavéria, France; thibault@volodalen.com; 3 Adams Centre for High Performance, Faculty of Health, Engineering, Computing and Science, School of Health, Sport and Human Performance, University of Waikato, Tauranga 3116, New Zealand; kim.hebert-losier@waikato.ac.nz; 4 Department of Sports Science, National Sports Institute of Malaysia, Kuala Lumpur 7102, Malaysia

**Keywords:** running, endurance, foot strike pattern, biomechanics, asymmetry

## Abstract

Close to 90% of recreational runners rearfoot strike in a long-distance road race. This prevalence has been obtained from North American cohorts of runners. The prevalence of rearfoot strikers has not been extensively examined in an Asian population of recreational runners. Therefore, the aim of this study was to determine the prevalence of rearfoot, midfoot, and forefoot strikers during a long-distance road race in Asian recreational runners and compare this prevalence to reported values in the scientific literature. To do so, we classified the foot strike pattern of 950 recreational runners at the 10 km mark of the Singapore marathon (77% Asian field). We observed 71.1%, 16.6%, 1.7%, and 10.6% of rearfoot, midfoot, forefoot, and asymmetric strikers, respectively. Chi-squared tests revealed significant differences between our foot strike pattern distribution and those reported from North American cohorts (*P* < 0.001). Our foot strike pattern distribution was similar to one reported from elite half-marathon runners racing in Japan (Fisher exact test, *P* = 0.168). We conclude that the prevalence of rearfoot strikers is lower in Asian than North American recreational runners. Running research should consider and report ethnicity of participants given that ethnicity can potentially explain biomechanical differences in running patterns.

## 1. Introduction

Even though each individual adopts a unique global natural running pattern, runners are typically classified in one of three discrete categories depending on their preferred foot strike pattern. Indeed, a runner is either categorized as a: (1) rearfoot striker when the initial contact of the foot with the ground is made on the heel or rear third part of the sole; (2) midfoot striker when the heel and toes contact the ground simultaneously; or (3) forefoot striker when the initial contact of the foot with the ground is made on the forefoot or front half of the sole [1]. These different foot strike patterns involve different neuromuscular activation patterns [2] and biomechanical strategies [3,4]. Furthermore, the likelihood of certain types of running-related injuries has been reported to depend on foot strike pattern [5,6,7]; with hip and knee injuries more common in rearfoot strikers, and ankle and foot injuries more common in forefoot strikers. The change in the relative risk of running-related injuries can be associated with the redistribution of loads on the basis of foot strike pattern [8,9,10,11].

The scientific evidence is inconclusive regarding the effect of foot strike pattern on running economy and performance. Most studies are observational in nature and report a higher proportion of non-rearfoot strikers in higher-performing runners [1,5]. These observational findings, along with the introduction of Pose [12] and Chi [13] running, have led to several runners adopting non-habitual forefoot strike patterns. Acute changes in natural foot strike patterns, however, can increase the energetic cost of running [14]. In the short and long-term, interventions to change a rearfoot strike pattern to a midfoot or forefoot one does not lead to a significant improvement in running economy [15,16]. Transitioning away from a rearfoot pattern, however, can decrease the energy required per unit of ground reaction force [15], shorten stride length [16], and increase stride frequency [16]. Decreasing stride length and transitioning to a non-rearfoot strike pattern may reduce certain risk factors for running-related injuries [17]. In certain cases, foot strike pattern modification can be a desirable intervention for the prevention of injuries or the rehabilitation of injured runners.

The prevalence of recreational runners that rearfoot strike during a long-distance road race is close to 90% [18,19]. The prevalence of rearfoot strikers is lower (~80%) in sub-elite runners [20], and lowest in elite runners [1,21,22]. One study reported that elite runners mostly midfoot and forefoot strike [21], although these observations stemmed from runners competing in 1500 m to marathon events. More recent studies on the topic report that the majority of elite runners rearfoot strike [1,22]. Approximately 75% of elite runners were rearfoot strikers at the 2004 Sapporo International Half Marathon in Japan [1], and more than 54% of marathoners were rearfoot strikers at the 2017 IAAF World Championships [22]. Two factors can explain the smaller proportion of rearfoot strikers observed by Hasegawa et al. [1] and Hanley et al. [22] compared to the 90% reported by Kasmer et al. [18] and Larson et al. [19]. First, Hasegawa et al. [1] and Hanley et al. [22] examined elite rather than recreational runners. Several studies have linked foot strike pattern with performance in road races and report a higher prevalence of midfoot and forefoot strikers in faster runners [1,18,20]. One second explanation to the smaller proportion of rearfoot strikers relates to ethnicity. Hasegawa et al. [1] examined a mostly Asian population of runners (even though it is not ensured due to the international nature of the event), whereas Kasmer et al. [18] and Larson et al. [19] observed foot strike patterns in a mostly North American cohort. In fact, the natural running pattern of individuals has been reported to differ between different ethnic groups [23,24].

The purpose of this study was to determine the prevalence of rearfoot, midfoot, and forefoot strikers during a long-distance road race in a predominantly Asian population of recreational runners. We hypothesized that the prevalence of rearfoot strikers in a predominantly Asian population of recreational runners would be lower than the prevalence of rearfoot strikers sourced from a predominantly North American based cohort of recreational runners.

## 2. Materials and Methods

### 2.1. Subjects

Nine hundred and fifty runners were videotaped at the 10 km mark (position 51 to 1000) of the 2015 Standard Chartered Singapore Marathon (Singapore, December 6) and classified according to their foot strike pattern. The nationality of the 51st to 1000th finisher was predominantly Asian (77%) according to the official race results. On this basis, we propose that the runners videotaped at the 10 km mark represented a mostly Asian cohort (77% Asian field) of recreational runners (finishing time range: 03:12:24 to 04:42:32, median: 04:15:49, average: 04:12:00 ± 00:22:22). The first 50 runners were not taken into account because they were considered elite and sub-elite (finishing time range: 02:17:26 to 03:11:30, average: 02:48:49 ± 00:18:40) and did not represent a predominantly Asian cohort (62% non-Asian runners). The study received approval from the Institutional Review Board of our institution (ISNRP: 26/2015).

### 2.2. Procedure

Runners were filmed using a Sony Handycam (HDR-PJ660, Sony Thai Co., Ltd., Bangkok, Thailand) digital video camera (50 Hz) with an actual focal length of 2.9 to 34.8 mm (35-mm equivalent focal length of 26.8 to 321.6 mm). The camera was securely mounted atop a 40-cm high tripod, 50 cm from the side of the road, and oriented such that the passing runners were filmed in the sagittal plane. The filming location represented a straight and level portion of the race to limit the influence of change in elevation on running patterns. The race course was about the width of a two-lane road. Therefore, the distance between each runner and the camera varied, but was sufficient for foot strike classification. The sex of the 950 marathon runners was determined from the video recordings.

Three foot strike patterns were considered and defined according to Hasegawa et al. [1]. More explicitly, forefoot strike (FFS) was defined as a foot strike in which the initial contact of the foot with the ground was the forefoot or front half of the sole and with no heel contact at foot strike. A midfoot strike (MFS) was defined as a foot strike in which the heel and toes contacted the ground simultaneously. A rearfoot strike (RFS) was defined as a foot strike in which the first contact of the foot with the ground was made on the heel or rear third part of the sole and with no midfoot or forefoot contact at foot strike. For each runner, both right and left foot strikes were classified.

From the 950 runners, five right and five left foot strikes were missing because these foot strikes were obscured (e.g., behind another runner) or clipped off at the edge of the video frame. We removed the data from these 10 runners during our statistical analyses, leading to a final sample size of 940 runners. An additional classification of split strike (SS) was used to identify runners exhibiting an asymmetry between left and right foot strikes [19]. The foot strike pattern of each runner was determined using a frame-by-frame analysis performed in Apple QuickTime Player. A single researcher with more than five years of experience in foot strike classification completed the analysis. The reliability of foot strike classification has been shown to be almost perfect between different raters (agreement > 99%) [18,25], indicating that a single rater is sufficient for accurate and generalizable foot strike data.

### 2.3. Statistical Analysis

The prevalence of each foot strike was calculated for our entire population (*n* = 940) and for each sex (*n* = 844 males, *n* = 96 females). We compared the foot strike distribution obtained for our sample to those reported by Kasmer et al. [18] and Larson et al. [19] using Chi-squared (χ^2^) tests without sex distinction because the information was not available in these two papers. The foot strike distribution obtained for our sample was compared to those reported by Hasegawa et al. [1] using Fisher exact tests given that some of the expected frequencies were less than five. As Hasegawa et al. [1] reported data from a mixture of left and right sides without addressing asymmetry, we included only symmetrical runners (*n* = 840) when contrasting our findings to those from Hasegawa et al. [1]. The expected frequencies used during Chi-squared and Fisher exact tests were based on those reported by Kasmer et al. [18], Larson et al. [19], and Hasegawa et al. [1].

The relationship between foot strike classification and sex for all runners was explored in our data set using Fisher exact tests given that one frequency was less than five. We also investigated differences in the position of individuals in the race between the four foot strike classifications (RFS, MFS, FFS, and SS) using a nonparametric Kruskal–Wallis test. Finally, we evaluated the prevalence of rearfoot, midfoot, and forefoot strikers as a function of the position of individuals in the race by grouping runners into groups of 100 consecutive participants and computing linear least square regressions together with their respective coefficient of determination (*R^2^*). Statistical analyses were done using customized scripts in R 3.5.0 (The R Foundation for Statistical Computing, Vienna, Austria) and Python 3.6.2 (Python Software Foundation, Beaverton, OR, USA). Level of significance was set at α ≤ 0.05.

## 3. Results

The 940 runners that were classified according to their foot strike pattern at the 10 km mark of the Singapore marathon are shown in Table 1. Chi-squared tests indicated that our observed foot strike distribution differed significantly from those reported by Kasmer et al. [18] (χ^2^ = 305.7, degrees of freedom = 3, *P* < 0.001) and Larson et al. [19] (χ^2^ = 112.8, degrees of freedom = 3, *P* < 0.001).

Amongst our 100 runners demonstrating a SS pattern at the 10 km mark of the Singapore marathon, 65 were rearfoot-right and midfoot-left, 29 were rearfoot-left and midfoot-right, 2 were rearfoot-right and forefoot-left, 1 was rearfoot-left and forefoot-right, 1 was midfoot-right and forefoot-left, and 2 were midfoot-left and forefoot-right. Prevalence of rearfoot, midfoot, and forefoot strikers excluding SS runners (*n* = 840) at the 10 km mark of the marathon are shown in Table 2. Fisher exact tests indicated that our observed foot strike distributions did not significantly differ from those reported by Hasegawa et al. [1] (all: *P* = 0.168, males: *P* = 0.170, and females: *P* = 0.918).

**Figure 1:**
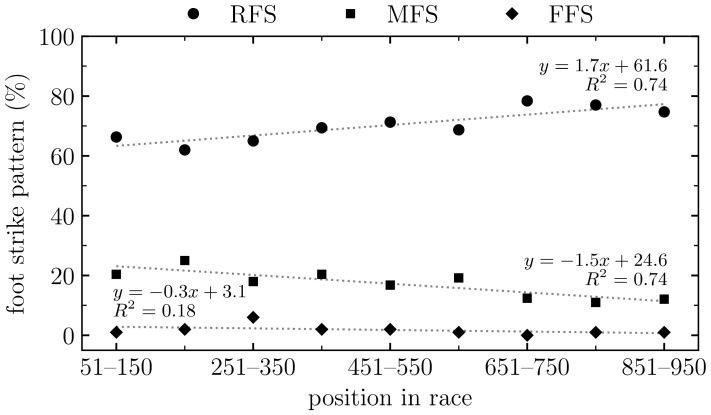
Prevalence of rearfoot strike (RFS), midfoot strike (MFS), and forefoot strike (FFS) patterns by position of individuals in race (groups of 100 runners) at the 10 km mark of the Singapore marathon. Dashed lines depict linear least square regressions together with the respective coefficient of determination (*R*^2^).

Fisher exact test revealed no significant difference in our prevalence of RFS, MFS, FFS, and SS between males and females at the 10 km mark (*P* = 0.135). The Kruskal–Wallis test revealed a significant difference between position of individuals in race and foot strike classification (χ^2^ = 19.0, degrees of freedom = 4, *P* < 0.001). Forefoot strikers tended to be faster, followed by midfoot, split, and rearfoot strikers. The prevalence of RFS, MFS, and FFS by position of individuals in the race (groups of 100 runners) is depicted in Figure 1. Split strikers represented, on average, 10.6 ± 1.3% of the runners when participants were grouped by 100 according to their rank.

## 4. Discussion

In accordance with our hypothesis, we obtained a lower prevalence of rearfoot strikers in an Asian population of recreational runners compared to the reported 90% in predominantly North American based cohorts. These findings indicate that ethnicity potentially explains biomechanical differences in running patterns between individuals, reinforcing the importance for researchers to report and consider ethnicity as a factor in running studies.

Available published data on foot strike patterns of North American recreational runners during long-distance road races (half-marathon and marathon) report that 90% of runners rearfoot strike [5,6]. In our study, the prevalence of runners that were rearfoot striking at the 10-km mark of the Singapore marathon differed significantly from those reported in both previous studies (*P* < 0.001). The average (04:12:00 ± 00:22:22) marathon finishing time of our 950 runners was greater than the average time reported by Larson et al. [19] (3:57:31 ± 00:34:17) likely due the hot and humid environmental conditions in Singapore. However, our study identified that rearfoot strikers (71.1% and 79.5% when excluding split strikers) were less common in our sample comprised of mostly Asian recreational runners (77% Asian field) than reported by Kasmer et al. [18] (Milwaukee Lakefront Marathon, Milwaukee, Wisconsin, USA) and Larson et al. [19] (Manchester City Marathon in Manchester, New Hampshire, USA). We propose that the ethnicity of our population of runners explains some of the discrepancy between the prevalence of rearfoot strikers we observed here and those reported by Kasmer et al. [18] and Larson et al. [19]. Nevertheless, rearfoot striking was still the most prevalent foot strike pattern in our population of predominantly Asian recreational runners.

The prevalence of rearfoot strikers reported in Hasegawa et al. [1] differed significantly from those reported in Larson et al. [19]. In addition, even though Hasegawa et al. [1] analyzed an elite population of runners, which was probably mostly comprised of Asian runners due to the location of the event (Japan), our prevalence of foot strike patterns (excluding SS runners, RFS = 79.5%, MFS = 18.6%, and FFS = 1.9%) did not differ significantly from those reported by Hasegawa et al. [1] (RFS = 74.9%, MFS = 23.7%, and FFS = 1.7%). Therefore, the elite character of the runners in the study of Hasegawa et al. [1] was probably not the only explanation behind the observed significant difference in foot strike pattern prevalence when compared to Larson et al. [19] (RFS = 94.4%, MFS = 3.6%, and FFS = 1.9%). Moreover, even though Lieberman et al. [26] observed that barefoot Kalenjin runners were forefoot strikers, Pontzer et al. [24] observed no forefoot, mostly midfoot, and several rearfoot strikers in barefoot Hadza runners. Furthermore, Hatala et al. [27] observed mostly rearfoot strikers in barefoot Daasanach runners. Altogether, these findings reinforce our current ones that ethnicity plays a role in the prevalence of rearfoot strikers in runners.

In agreement with previous studies [1,18,20], our study observed a significant difference between the position in race and foot strike pattern prevalence (*P* < 0.001). More specifically, we observed an increase in the prevalence of rearfoot strikers (*R*^2^ = 0.74) together with a decrease in the prevalence of midfoot strikers (*R*^2^ = 0.74) as in-race position worsened (i.e., in the slower runners, Figure 1). The faster runners in our study were more likely to be midfoot strikers, as observed by Hasegawa et al. [1], Kasmer et al. [18], and Kerr et al. [20], but not by Larson et al. [19], which may be due to their relatively smaller sample size (*n* = 286). On the other hand, we observed that the prevalence of forefoot strikers did not seem to be related to the position in race (*R*^2^ = 0.18), which can be explained by the small percentage of runners (1.8 ± 1.7% when grouped by 100 according to rank) that adopted a FFS pattern across performance levels. Moreover, we observed that the number of split strikers was constant across the examined in-race position bands (10.6 ± 1.3%).

Nevertheless, it is important to mention that although we classified runners in three different categories, foot strike pattern is actually a continuum [28,29]. Therefore, each discrete category encompasses a wide variation of running patterns, not only in terms of which part of the foot first contacts the ground, but also in terms of several other biomechanical parameters such as the leg flexion or ankle angle at contact. Different foot strike patterns involve different neuromuscular activation patterns [2], biomechanical strategies [3,4], and running-related injury patterns [5,6,7]. During a marathon, rearfoot and midfoot strikers have been shown to exhibit higher tibial shocks at foot strike compared to forefoot strikers, suggesting a higher loading impact in RFS and MFS than FFS [3]. Plantar loads are relatively greater in the rearfoot and midfoot regions in RFS, and in the forefoot region in MFS and FFS [4]. Changes in the magnitude and location of loads according to foot strike pattern are important to consider in the management of runners.

A few limitations to the present study exist. The frame rate was set to 50 Hz, a value which is smaller than the ones used in previous studies [1,18,19]. As the duration of the ground contact phase is reported to vary between 0.29 and 0.21 s for running speeds ranging from 10 to 14 km/h [30], we believe that a 50 Hz frame rate (0.02 s per frame) is sufficient to detect the part of the foot that first makes contact with the ground at marathon running speeds. Furthermore, another factor that was not considered in this study is footwear. Variance in the prevalence of runners presenting with a rearfoot strike might result from running shoe characteristics, where more minimal shoes (i.e., lower mass, heel height, and heel-to-toe drop) are typically associated with fewer rearfoot strikers compared to more cushioned shoes [31]. Indeed, Larson [32] observed a relatively low prevalence of rearfoot strikers in barefoot (20.7%) and minimally shod (47.6%) runners 350 m into the New York City Barefoot Run. It could be that footwear market and shoe preference of Asian runners are biased towards more minimal shoes. Shoe characteristics could not be accurately defined from our video footage, so no classification of footwear was attempted.

## 5. Conclusions

We conclude that the prevalence of rearfoot strikers in long-distance races is lower in Asian than North American recreational runners. These findings indicate that ethnicity should be considered and reported as a factor in running research given that it can potentially explain biomechanical differences in running patterns.

## Figures and Tables

**Table 1:** Number of foot strike patterns (count and %) observed in recreational runners at the 10 km mark of a marathon.

FSP	All			Males			Females		
	Singapore	Kasmer	Larson	Singapore	Kasmer	Larson	Singapore	Kasmer	Larson
RFS	668	1867	832	596	1073	176	72	792	75
	(71.1)	(93.7)	(88.9)	(70.6)	(92.5)	(85.9)	(75.0)	(95.3)	(92.5)
MFS	156	101	31	143	71	8	13	30	1
	(16.6)	(5.1)	(3.4)	(16.9)	(6.1)	(3.9)	(13.5)	(3.6)	(1.3)
FFS	16	11	17	12	7	3	4	4	1
	(1.7)	(0.55)	(1.8)	(1.4)	(0.60)	(1.5)	(4.2)	(0.48)	(1.3)
SS	100	14	55	93	9	18	7	5	4
	(10.6)	(0.70)	(5.9)	(11.0)	(0.78)	(8.7)	(7.3)	(0.60)	(5.0)

Note. FSP: foot strike pattern, RFS: rearfoot strike, MFS: midfoot strike, FFS: forefoot strike, SS: split strike. References: Kasmer et al. [18], Larson et al. [19].

**Table 2:** Number of foot strike patterns (count and %) observed in recreational runners at the 10 km mark of a marathon.

FSP	All		Males		Females	
	Singapore	Hasegawa	Singapore	Hasegawa	Singapore	Hasegawa
RFS	668	212	596	184	72	28
	(79.5)	(74.9)	(79.4)	(74.2)	(80.9)	(80.0)
MFS	156	67	143	61	13	6
	(18.6)	(23.7)	(19.0)	(25.6)	(14.6)	(17.1)
FFS	16	4	12	3	4	1
	(1.9)	(1.4)	(1.6)	(0.2)	(4.5)	(2.9)

Note. FSP: foot strike pattern, RFS: rearfoot strike, MFS: midfoot strike, FFS: forefoot strike, SS: split strike. Reference: Hasegawa et al. [1].

## References

[1] Hasegawa H., Yamauchi T., Kraemer W.J. (2007). Foot strike patterns of runners at the 15-km point during an elite-level half marathon. J. Strength Cond. Res..

[2] Ahn A.N., Brayton C., Bhatia T., Martin P. (2014). Muscle activity and kinematics of forefoot and rearfoot strike runners. J. Sport Health Sci..

[3] Wei Z., Zhang Z., Jiang J., Zhang Y., Wang L. (2019). Comparison of plantar loads among runners with different strike patterns. J. Sports Sci..

[4] Ruder M., Jamison S., Tenforde A., Mulloy F., Davis I.S. (2019). Relationship of footstrike pattern and landing impacts during a marathon. Med. Sci. Sports Exerc..

[5] Daoud A.I., Geissler G.J., Wang F., Saretsky J., Daoud Y.A., Lieberman D.E. (2012). Foot strike and injury rates in endurance runners: A retrospective study. Med. Sci. Sports Exerc..

[6] Walther M., Reuter I., Leonhard T., Engelhardt M. (2005). Injuries and response to overload stress in running as a sport. Orthopade.

[7] Altman A.R., Davis I.S. (2016). Prospective comparison of running injuries between shod and barefoot runners. Br. J. Sports Med..

[8] Vannatta C.N., Kernozek T.W. (2015). Patellofemoral joint stress during running with alterations in foot strike pattern. Med. Sci. Sports Exerc..

[9] Rooney B.D., Derrick T.R. (2013). Joint contact loading in forefoot and rearfoot strike patterns during running. J. Biomech..

[10] Dos Santos A.F., Nakagawa T.H., Serrao F.V., Ferber R. (2019). Patellofemoral joint stress measured across three different running techniques. Gait Posture.

[11] Almonroeder T., Willson J.D., Kernozek T.W. (2013). The effect of foot strike pattern on achilles tendon load during running. Ann. Biomed. Eng..

[12] Arendse R.E., Noakes T.D., Azevedo L.B., Romanov N., Schwellnus M.P., Fletcher G. (2004). Reduced eccentric loading of the knee with the pose running method. Med. Sci. Sports Exerc..

[13] Dreyer D., Dreyer K. (2009). ChiRunning: A Revolutionary Approach to Effortless, Injury-Free Running, Revised and Fully Updated ed.

[14] Gruber A.H., Umberger B.R., Braun B., Hamill J. (2013). Economy and rate of carbohydrate oxidation during running with rearfoot and forefoot strike patterns. J. Appl. Physiol..

[15] Ekizos A., Santuz A., Arampatzis A. (2018). Short- and long-term effects of altered point of ground reaction force application on human running energetics. J. Exp. Biol..

[16] Craighead D.H., Lehecka N., King D.L. (2014). A novel running mechanic’s class changes kinematics but not running economy. J. Strength Cond. Res..

[17] Napier C., MacLean C.L., Maurer J., Taunton J.E., Hunt M.A. (2019). Kinematic correlates of kinetic outcomes associated with running-related injury. J. Appl. Biomech..

[18] Kasmer M.E., Liu X., Roberts K.G., Valadao J.M. (2013). Foot-strike pattern and performance in a marathon. Int. J. Sports Physiol. Perform..

[19] Larson P., Higgins E., Kaminski J., Decker T., Preble J., Lyons D., McIntyre K., Normile A. (2011). Foot strike patterns of recreational and sub-elite runners in a long-distance road race. J. Sports Sci..

[20] Kerr B.A., Beauchamp L., Fisher V., Neil R., Nigg B.M., Kerr B.A. (1983). Foot Strike Patterns in Distance Running. Biomechanical Aspects of Sport Shoes and Playing Surfaces, Proceedings of the International Symposium on Biomechanical Aspects of Sport Shoes and Playing Surfaces.

[21] Nett T. (1964). Foot plant in running. Track Tech..

[22] Hanley B., Bissas A., Merlino S., Gruber A.H. (2019). Most marathon runners at the 2017 IAAF World Championships were rearfoot strikers, and most did not change footstrike pattern. J. Biomech..

[23] Hébert-Losier K., Lussiana T., Tee C.C.L., Baca A., Wessner B., Diketmüller R., Tschan H., Hofmann M., Kornfeind P., Tsolakidis E. (2016). The running biomechanics and economy of south east asians: We do not all run the same. Proceedings of the 21st Annual Congress of the European College of Sport Science.

[24] Pontzer H., Suchman K., Raichlen D.A., Wood B.M., Mabulla A.Z.P., Marlowe F.W. (2014). Foot strike patterns and hind limb joint angles during running in hadza hunter-gatherers. J. Sport Health Sci..

[25] Murray L., Beaven C.M., Hébert-Losier K. (2018). Reliability of overground running measures from 2d video analyses in a field environment. Sports.

[26] Lieberman D.E., Venkadesan M., Werbel W.A., Daoud A.I., D’Andrea S., Davis I.S., Ojiambo Mang’Eni R., Pitsiladis Y. (2010). Foot strike patterns and collision forces in habitually barefoot versus shod runners. Nature.

[27] Hatala K.G., Dingwall H.L., Wunderlich R.E., Richmond B.G. (2013). Variation in foot strike patterns during running among habitually barefoot populations. PLoS ONE.

[28] Altman A.R., Davis I.S. (2012). A kinematic method for footstrike pattern detection in barefoot and shod runners. Gait Posture.

[29] Cavanagh P.R., Lafortune M.A. (1980). Ground reaction forces in distance running. J. Biomech..

[30] Lussiana T., Patoz A., Gindre C., Mourot L., Hébert-Losier K. (2019). The implications of time on the ground on running economy: Less is not always better. J. Exp. Biol..

[31] Squadrone R., Rodano R., Hamill J., Preatoni E. (2015). Acute effect of different minimalist shoes on foot strike pattern and kinematics in rearfoot strikers during running. J. Sports Sci..

[32] Larson P. (2014). Comparison of foot strike patterns of barefoot and minimally shod runners in a recreational road race. J. Sport Health Sci..

